# SCARB1 single nucleotide polymorphism (rs5888) is associated with serum lipid profile and myocardial infarction in an age- and gender-dependent manner

**DOI:** 10.1186/1476-511X-12-24

**Published:** 2013-03-05

**Authors:** Daiva Stanislovaitiene, Vaiva Lesauskaite, Dalia Zaliuniene, Alina Smalinskiene, Olivija Gustiene, Diana Zaliaduonyte-Peksiene, Abdonas Tamosiunas, Dalia Luksiene, Janina Petkeviciene, Remigijus Zaliunas

**Affiliations:** 1 Department of Ophthalmology, Medicine Academy, Lithuanian University of Health Sciences, Kaunas, Lithuania; 2 Intitute of Cardiology, Medicine Academy, Lithuanian University of Health Sciences, Kaunas, Lithuania; 3 Department of Cardiology, Medicine Academy, Lithuanian University of Health Sciences, Kaunas, Lithuania; 4 Faculty of Public Health, Medical Academy, Lithuanian University of Health Sciences, Kaunas, Lithuania

**Keywords:** Coronary artery disease (CAD), Myocardial infarction (MI), Scavenger receptor Class B Type 1 gene (SCARB1), Single nucleotide polymorphism (SNP)

## Abstract

**Background:**

Mutation in SCARB1 gene, exon 8 rs5888, has been associated with altered lipid levels and cardiovascular risk in humans though the results have been inconsistent. We analysed the impact of SCARB1 single nucleotide polymorphism (SNP) rs5888 with plasma lipid profile and association with coronary artery disease (CAD) in a Lithuanian population characterized by high morbidity and mortality from CAD and high prevalence of hypercholesterolemia.

**Methods:**

The study included 1976 subjects from a random sample (reference group) and an myocardial infarction (MI) group of 463 patients. Genotyping of SCARB1 (rs5888) was carried out using the real-time polymerase chain reaction method.

**Results/principal findings:**

Analysis of rs5888 C/T gene polymorphism in the reference group revealed that male TT genotype carriers (25–74 years) had significantly higher total cholesterol and triglyceride concentrations (5.70 mmol/l vs. 5.49 mmol/l; p = 0.036, and 1.70 mmol/l vs. 1.40 mmol/l, p = 0.023, respectively) than CT carriers and the oldest males (65–74 years) TT carriers had significantly higher high density lipoprotein cholesterol concentrations in comparison to heterozygous (1.52 mmol/l vs. 1.36 mmol/l, p = 0.033). The youngest female (25–44 years) TT genotype carriers had significantly lower low density lipoprotein cholesterol concentrations in comparison to C homozygous (2.59 mmol/l vs. 2.92 mmol/l, p = 0.023). The frequency of the SCARB1 TT genotype in the oldest male MI group (65–74 years) was significantly lower than in the corresponding reference group subjects (9.4% vs. 22.3%, p = 0.006). SCARB1 TT genotype was associated with decreased odds of MI in males aged 65–75 years (OR = 0.24, 95% CI 0.10-0.56, p = 0.001).

**Conclusions/significance:**

SCARB1 polymorphism is associated with lipid metabolism and CAD in an age- and gender- dependent manner. Analysis of SCARB1 SNP rs5888 C/T genotypes revealed an atheroprotective phenotype of lipid profile in older men and in young women TT genotype carriers in the reference group. SCARB1 TT genotype was associated with decreased odds of MI in aged men.

## Background

Coronary artery disease (CAD) is intimately associated with dyslipidaemias. Plasma lipid concentration is influenced by environmental and genetic factors. A number of variants in candidate genes have been implicated in the regulation of plasma lipid levels [1]. The scavenger receptor class B type I (SCARB1) is a key component in the reverse cholesterol transport pathway where it binds high density lipoprotein cholesterol (HDL-Chol) with high affinity and is involved in the selective transfer of lipids from HDL-Chol [2,3]. Moreover, SCARB1 is a multiligand receptor binding other native lipoproteins such as (i) low density lipoprotein (LDL); (ii) very low density lipoprotein (VLDL) [4]; and (iii) a variety of ligands, including modified (acetylated or oxidized) lipoproteins, anionic phospholipids and apoptotic cells [4,5]. These receptors that were initially found in cultured macrophages [6], are also highly expressed in the liver and in steroidogenic tissues [7]; their expression is regulated by a number of factors including estrogens [8-13], dietary cholesterol [14] and genetic variation [13,15]. The importance of SCARB1 in overall cholesterol metabolism and its antiatherogenic activity *in vivo* has been definitively established by SCARB1 gene manipulation in mice [6]. The role of SCARB1 on cholesterol metabolism in humans was analysed by studying the influence of SCARB1 gene variants on plasma lipid concentration [15-23]. Several polymorphic variants have been described in the human SCARB1 gene [16,19]. Mutation in SCARB1 gene, exon 8 rs5888, has been associated with altered lipid levels and cardiovascular risk in humans [19,21,24], however the results have been inconsistent [16,18,21-23].

In our study we analyzed the impact of SCARB1 SNP rs5888 with serum lipid profiles and association with CAD in a Lithuanian population characterised by high morbidity and mortality from CAD and a high prevalence of hypercholesterolemia [25,26].

## Results

Fasting serum lipid concentrations of the Lithuanian population (reference group) according to SCARB1 genotypes, gender and age are shown in Table 1. For men, the SCARB1 TT genotype was associated with higher means of fasting serum lipid concentrations. TT genotype carriers aged 25–74 years had a significantly higher total cholesterol concentration than heterozygous (p = 0.036) and higher than CC subjects (p = 0.050). In the age group 45–64 years and in all reference groups (25–74 years) TT genotype carriers had a significantly higher LDL-Chol than C homozygous (3.77 mmol/l vs. 3.48 mmol/l, p = 0.022 and 3.68 mmol/l vs. 3.48 mmol/l, p = 0.035, respectively). Only the oldest (65–74 years) TT subjects had significantly higher HDL-Chol concentrations in comparison to heterozygous (1.52 mmol/l vs. 1.36 mmol/l, p = 0.033). The youngest (25–44 years) TT genotype carriers had significantly higher serum TG concentrations than heterozygous (1.70 mmol/l vs. 1.40 mmol/l, p = 0.023). The same regularity was observed in the total reference group (25–74 years) (1.57 mmol/l vs. 1.43 mmol/l, p = 0.037). There were no significant differences in fasting lipid serum concentrations between CC and CT genotype carriers with an exception for the TG concentration in males aged 25–44 years (1.68 mmol/l and 1.40 mmol/l, p = 0.003).

**Table 1:** Fasting serum lipids concentrations of the Lithuanian population (reference group) according to SCARB1 genotypes (Mean (Standard Deviation)) by gender and age

**Characteristics**	***SCARB1 *genotypes**	**Men**				**Women**			
		**25-44 years**	**45-64 years**	**65-74 years**	**All**	**25-44 years**	**45-64 years**	**65-74 years**	**All**
		**N = 168**	**N = 590**	**N = 144**	**N = 902**	**N = 260**	**N = 652**	**N = 162**	**N = 1074**
TChol, mmol/l	CC	5.27 (1.00)	5.52 (1.15)	5.66 (0.98)	5.49 (1.05)	4.85 (0.89)	5.85 (1.07)	6.02 (1.04)	5.63 (0.98)
	CT	5.20 (1.02)	5.58 (1.01)	5.43 (0.98)	5.49 (1.05)	4.89 (0.88)	5.81 (1.08)	6.20 (1.01)	5.65 (1.14)
	TT	5.5 (1.01)	5.76 (1.09)	5.75 (0.99)	5.70* (1.12)	4.75 (0.83)	5.96 (1.08)	6.41 (1.08)	5.64 (1.05)
	Total	5.27 (1.04)	5.59 (0.97)	5.57 (0.96)	5.53 (0.90)	4.86 (0.81)	5.82 (1.02)	6.19 (1.02)	5.64 (0.98)
LDL-Chol, mmol/l	CC	3.32 (0.92)	3.48 (1.00)	3.65 (0.92)	3.48 (1.05)	2.92 (0.79)	3.68 (1.07)	3.79 (0.96)	3.51 (0.98)
	CT	3.37 (0.93)	3.58 (1.01)	3.47 (0.90)	3.53 (1.05)	2.87 (0.77)	3.67 (1.08)	4.02 (1.01)	3.52 (0.91)
	TT	3.56 (0.96)	3.77^**^ (0.99)	3.58 (0.93)	3.68^**^ (1.00)	2.59^**^ (0.77)	3.62 (0.98)	4.22** (0.96)	3.49 (0.92)
	Total	3.38 (0.91)	3.58 (0.97)	3.55 (0.96)	3.54 (0.90)	2.84 (0.81)	3.67 (1.02)	3.98 (1.02)	3.51 (0.98)
HDL-Chol, mmol/l	CC	1.21 (0.38)	1.37 (0.43)	1.42 (0.33)	1.35 (0.35)	1.40 (0.30)	1.51 (0.31)	1.55 (0.30)	1.49 (0.39)
	CT	1.32 (0.37)	1.39 (0.34)	1.36 (0.33)	1.38 (0.42)	1.47 (0.33)	1.49 (0.36)	1.48 (0.34)	1.48 (0.45)
	TT	1.21 (0.43)	1.35 (0.40)	1.52^*^ (0.35)	1.36 (0.37)	1.50 (0.32)	1.47 (0.39)	1.46 (0.06)	1.48 (0.39)
	Total	1.27 (0.39)	1.38 (0.49)	1.42 (0.36)	1.36 (0.30)	1.45 (0.32)	1.49 (0.26)	1.50 (0.38)	1.48 (0.33)
TG, mmol/l	CC	1.68 (0.54)	1.51 (0.86)	1.29 (0.59)	1.50 (0.70)	1.33 (0.49)	1.44 (0.61)	1.50 (0.67)	1.42 (0.59)
	CT	1.40^***^ (0.56)	1.46 (0.85)	1.32 (0.57)	1.43 (0.84)	1.36 (0.44)	1.47 (0.72)	1.52 (0.67)	1.45 (0.68)
	TT	1.70^*^ (0.58)	1.57 (0.80)	1.43 (0.58)	1.57^*^ (0.75)	1.29 (0.45)	1.50 (0.69)	1.61 (0.66)	1.47 (0.66)
	Total	1.54 (0.52)	1.50 (0.73)	1.33 (0.60)	1.48 (0.60)	1.34 (0.48)	1.46 (0.77)	1.54 (0.64)	1.44 (0.66)

Data adjusted by age and body mass index (BMI) in each age group. *Abbreviations:* TChol – total cholesterol; LDL-Chol – low density lipoprotein cholesterol; HDL-Chol – high density lipoprotein cholesterol; TG – triglyceride.

* - means significantly different (p <0.05) between TT and CT.

** - means significantly different (p < 0.05) between TT and CC.

*** - means significantly different (p < 0.05) between CT and CC.

In women, only the concentration of serum LDL-Chol was significantly associated with SCARB1 C/T genotypes. The youngest women (25–44 years) TT genotype carriers had significantly lower LDL-Chol in comparison to C homozygous (2.59 mmol/l vs. 2.92 mmol/l, p = 0.023) and lower than CT subjects (2.59 mmol/l vs. 2.87 mmol/l, p = 0.054). In contrast, the oldest TT genotype carriers had significantly higher LDL-Chol than C homozygous (4.22 mmol/l vs 3.79 mmol/l, p = 0.045).

**Figure 1:**
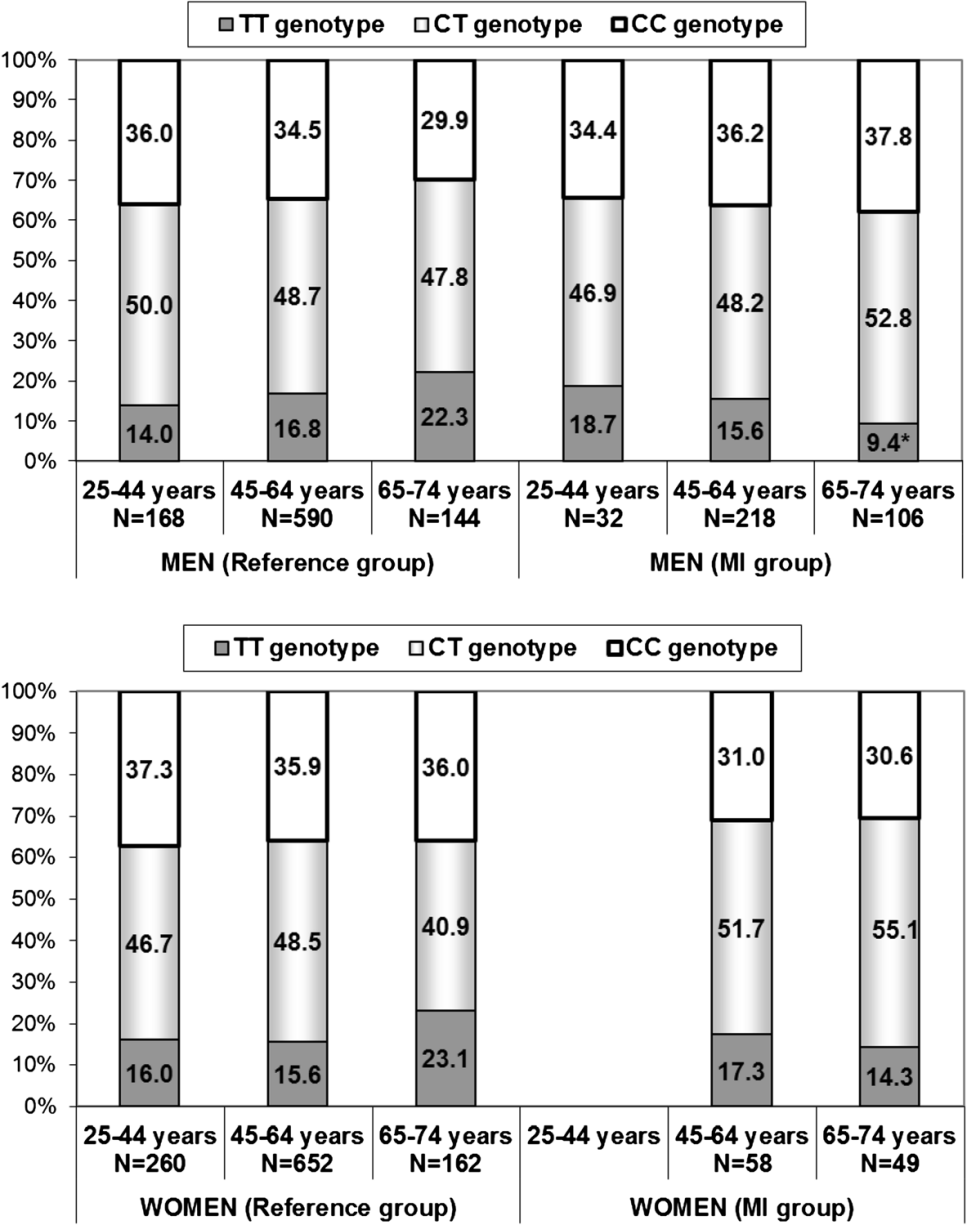
**Distribution (%) of *SCARB1 *C/T (rs5888) genotypes in reference and myocardial infarction (MI) groups.** * p = 0.006 in comparison with a reference group. There are no data on the frequency of SCARB1 genotypes in women with MI at the age of 25–44 years due to the small number of study subjects in this group (n = 5).

The frequency of SCARB1 (rs5888) T allele was 36.2% in the reference group and 40.0% in the MI group. The frequency of SCARB1 genotypes by gender and age is presented in Figure 1. There was no significant differences in SCARB1 C/T genotype distribution between men and women in both reference and MI groups. There was a tendency of increased frequency of TT genotype with age in men and in women from reference groups. The frequency of TT genotype was lowest in the 25–44 year group and highest in 65–74 year group (in men - 14.0% vs. 22.3%, p = 0.06, and in women - 16.0%, vs. 23.1%. p = 0.07, respectively).

The frequency of the SCARB1 TT genotype in the oldest male MI group (65–74 years) was significantly lower than in the corresponding reference group subjects (9.4% vs. 22.3%, p = 0.006, respectively). Multivariable binary logistic regression analysis was performed to identify the effect of SCARB1 genotype TT vs. SCARB1 genotype CC and CT on the risk of MI; separately for men and women in all three age groups (25–44, 45–64, 65–74). The results of multivariable logistic regression analysis showed that SCARB1 TT genotype was associated with decreased odds of MI only in men aged 65–74 years (OR = 0.24, 95% CI 0.10-0.56, p = 0.001) compared with the reference group of men aged 65–74 years (data were adjusted by age and BMI).

## Discussion

The impact of SCARB1 SNP rs5888 on serum lipid profiles and association with CAD in a Lithuanian population were studied. According to our data the frequency of minor T allele in the reference and MI groups was 36.2% and 40.0%, respectively. In Framingham study, where nearly all subjects were Caucasian, the frequency of SCARB1 T allele was 48.6% [18]. Whereas in Chinese population T allele is more recent [27,28]. It’s frequency was 21.7% in Bai Ku Yao and 26.3% in Han Chinese [27].

The data on the impact of SCARB1 SNP rs5888 on the serum lipid profile in human populations are inconsistent (Table 2). Previous studies reported no influence on serum lipid profile [15,17,21], while others proved a beneficial T allele effect on anti-atherogenic lipid profile [16,18-20,22,23] or even diminished CAD risk [19,21].

**Table 2:** Effect of SCARB1 gene exon 8 SNP rs5888 on serum lipid profile in human populations

**Study subjects**	**Effect of *SCARB1 *gene exon 8 SNP rs5888 (minor T allele carriers)**	**Reference**
White Americans with Coronary artery disease (n = 371);	No associations with lipid levels.	McCarthy et al.; 2003 [17]
Age of 45 yrs and older		
White Americans – Amish population (n = 919). Age of 18 yrs and older.	**↑HDL-Chol levels in women less than 50 years.**	Roberts et al.; 2007 [22]
	No associations with lipid concentrations in men.	
White Caucasians - population of Geneva, Switzerland (n = 1756). Aged 35–74 yrs.	**↑ HDL-Chol level and HDL-Chol/LDL-Chol ratio in men CT + TT vs CC carriers aged 55–74 yrs.**	Morabia et al.; 2004 [19]
Postmenopausal Caucasian women (n = 689)	**↑HDL-Chol among estrogen users.**	Richard et al.; 2005 [20]
North Americans/Framingham, USA (n = 2650).	**↑HDL-Chol, ↓LDL-Chol.**	Osgood et al.; 2003 [18]
Aged 26–79 yrs.	**Associated with an ↑ in HDL particle diameter in both men and women.**	
Healthy white Europeans – Spanish population (n = 489).	**↓LDL-Chol in women.**	Acton et al.; 1999 [16]
Mean age: women 36 ± 12 yrs; in men 39 ± 19 yrs.	No associations with lipids concentration in men	
Hypercholesterolemic and normolipidemic Brasilian individuals (n = 332).	No relation with lipid levels.	Cerda et al.; 2010 [15]
Aged 29–81 yrs.	**↑ response to atorvastatin in women.**	
Canarian/Spanish population (n = 619).	No association with lipid levels.	Rodriguez-Esparragon et al.; 2005 [21]
Aged 25 to 79 yrs.	**↓Coronary heart disease risk for men.**	
Healthy Spanish men population (n = 59).	No difference in fasting lipid concentrations by genotype.	Tanaka et al.; 2007 [23]
Aged 18–49 yrs.	**↓ postprandial TG response in the smaller, partially catabolized lipoprotein fraction reflects an antiatherogenic phenotype.**	
Two Chinese populations: Guangxi Bai Ku Yao, Han (n = 1183).	**↓HDL-Chol** than in CC or CT carriers.	Wu et al.; 2012 [27]
Aged 16 to 80 yrs.		
Two Chinese populations: Mulao, Han (n = 1608).	**↓HDL-Chol in Mulao females** compared with CC or CT carriers;	Wu et al.; 2012 [28]
Aged 16 to 86 yrs.	**↓HDL-Chol in Han males** compared with CC subjects;	
	**↑TG in Han subjects** compared to CC or CT.	

**↑ -** increases; **↓ -** decreases.

The effect of SCARB1 SNP rs5888 depended on age [19,22] and gender [15,16,19,21]. According to our data the effect of SCARB1 minor allele T on serum lipid profile depended on gender and age as well. In our study, male TT genotype carriers had higher LDL-Chol level than C allele carriers. The difference achieved a significant level between T and C homozygous subjects in the group aged 45–64 years as well as in all reference groups. Beneficial SCARB1 TT genotype effect on HDL-Chol was found only in the oldest (65–74 years) male group as opposed to younger men.

An atheroprotective T allele effect on HDL-Chol in older men was reported by Morabia et al. as well [19]. Contrary to men, a beneficial T allele effect on HDL-Chol level was reported in women younger than 50 years [22] or among postmenopausal estrogen users [20]. We did not find a T allele effect on HDL-Chol level in women, while a beneficial TT genotype effect on lipid profile emerged in the youngest female group. Women TT carriers aged 25–44 years had significantly lower LDL-Chol levels than C allele carriers. These data are concordant with the results from the healthy Spanish subjects investigation [16]. According to our data the TT genotype effect on LDL-Chol level changed during aging in women. There was no significant influence of SCARB1 C/T genotypes on the serum LDL-Chol level in the female group aged 45–65 years, while in the older age group we found the same regularity as in men. This suggests that women TT carriers older than 65 years had significantly higher LDL-Chol levels than CC genotype subjects.

Decreased HDL-Chol, raised LDL-Chol and TG levels are well known risk factors for the development of CAD and atherosclerosis [29,30]. As discussed above, the atheroprotective phenotype of lipid profile was found in older men and in young women TT genotype carriers. The former had higher HDL-Chol levels while the latter had lower LDL-Chol levels in comparison to C allele carriers. Thus our data indicate that the SCARB1 SNP rs5888 TT genotype has a varying effect on the phenotypic lipid profile and that this is dependent on both age and gender. Moreover, analysis of SCARB1 SNP rs5888 C/T genotypes in a reference group and MI patients revealed a protective effect of the TT genotype in aged males. We observed the lower frequency of the SCARB1 TT genotype in male MI patients aged 65–74 years as compared to the corresponding reference group subjects (9.4% and 22.3% respectively, p = 0.006).

Finally, the men TT carriers aged 65–74 years had significantly lower MI risk (OR = 0.24; p = 0.001), while Rodríguez-Esparragón et al. confirmed that the higher CAD risk is associated with SCARB1 (C1050T) CC genotype in males [21]. Atherogenic lipid phenotype had TT allele carriers in men aged 25–44 years (they have higher TG level), middle aged men and aged women (both of the latter have higher LDL-Chol level), but we did not find an association between TT genotype and higher risk of MI in these specific age-gender groups.

According to our data SCARB1 rs5888 SNP had influence not only on the level of HDL-Chol, but on the TG and LDL-Chol levels as well. SCARB1 was identified as the first molecularly well defined and physiologically important HDL receptor [2], but it is also a multiligand receptor participating not only in HDL-Chol but in other plasma lipoprotein metabolism. This was demonstrated by experiments with transgenic mice with liver-specific overexpression of murine SCARB1. On a chow diet SCARB1 transgenic mice have decreased HDL-Chol, apoA-I, and apoA-II levels; serum TG, LDL-Chol, VLDL and LDL apoB were also decreased, compared with control mice [31]. Animal studies carried out on rat demonstrated that immunodetectable SCARB1 is most highly expressed in the adrenal gland, ovary, and liver and its expression depends on estrogens [9]. In the same study it was demonstrated that high-dose estrogen treatment reduced SCARB1 in the liver and increased SCARB1 in the adrenal gland and corpus luteal cells of the ovary. Such estrogen-induced increase in SCARB1 was accompanied by enhanced *in vivo* uptake of lipid from HDL. Prolonged adrenal stimulation by adrenocorticotropic hormone in rats and mice decreased hepatic SCARB1 protein expression and it lead to the increase of plasma HDL-Chol level [32]. The down-regulation by estrogen effect on SCARB1 was demonstrated in humans as well [33]. It was shown that females have significantly lower levels of hepatic SCARB1 expression in comparison to males. Chiba-Falek O. et al. proposed that the protection afforded by estrogen in younger women may be limited to women with a certain SCARB1 genotype [32]. Rodriguez-Esparragon et al. [21] demonstrated that peripheral blood mononuclear cells taken from TT carriers had higher expression of SCARB1 mRNA in comparison to C allele carriers. In spite of this, the mechanism of observed SCARB1 SNP rs5888 influence on lipid profile remains obscure, as the function of SNP rs5888 is not finally elucidated. This SNP, despite being in an exon does not lead to a change in the amino acid sequence and does not appear to be a functional mutation [16,19]. This SNP might be in linkage disequilibrium with a functional mutation at a relevant locus [16], or its effect might be generated through another mechanism such as some splicing regulatory role [19].

### The study has some limitations

Most of patients with MI were receiving lipid lowering treatment. Thus we were not able to present data on the impact of SCARB1 SNP rs5888 on lipid profile in these patients. There are no data on the frequency of SCARB1 genotypes in women with MI at the age of 25–44 years as well. Data were not analyzed due to the small number of study subjects in this group (n = 5).

In conclusion, our results suggest that the SCARB1 rs5888 polymorphism is associated with serum lipid profile and MI in an age- and gender-dependent manner. Analysis of SCARB1 SNP rs5888 C/T genotypes revealed an atheroprotective phenotype of lipid profile in old men and in young women TT genotype carriers. TT genotype was associated with decreased odds of MI in aged men. Further studies are required to explain the biological basis of the SCARB1 SNP rs5888 effect on lipid profile.

## Materials and methods

### Study design and sample

The cross-sectional health survey was carried out in 2006–2008 in Kaunas which is the second largest city in Lithuania, and in five regions randomly selected from the northern, southern, eastern, western and central parts of Lithuania. The random samples of inhabitants aged 25 to 74 years and stratified by gender, age and place of residence were randomly selected from the Lithuanian population register. The eligible initial sample cohort included 3442 individuals. From these, 2079 (60.4%) underwent a health examination. Individuals who reported the current use of lipid lowering medication (N = 90) and women who reported still taking hormonal replacement therapy (N = 13) were excluded from analysis. So, in the study 1976 subjects were presented as our reference group.

Those who had suffered myocardial infarction (MI) included 468 patients aged 28–74 years with an acute MI or with a history of MI. All MI patients were admitted to the Cardiology department of the Lithuanian University of Health Sciences with a diagnosis of an acute MI made on the basis of prolonged chest pain, elevated troponin I serum levels, ST and T wave abnormalities on ECG, and typical wall motion abnormalities on the baseline 2-D echocardiogram; old MIs were diagnosed on anamnestic and clinical data of the patient and official medical recordings. From these, only 5 women were aged 25–44 years, so due to this small number of cases in this age group they were excluded from analysis. Patients with MI were receiving lipid lowering treatment.

Finally, this article presents the findings for 2439 subjects: 1976 subjects we presented as a reference group (902 men and 1074 women) and the MI group included 463 patients (356 men and 107 women).

### SCARB1 genotyping

For DNA extraction blood samples were collected in ethylenediaminetetraacetic (EDTA) tubes at the health check. DNA was extracted from peripheral blood leukocytes with a kit (NucleoSpin Blood L Kit; Macherey & Nagel, Düren, Germany) according to the instructions. Single-nucleotide polymorphism (SNP) of SCARB1 (rs5888) gene was assessed using commercially available genotyping kit C_7497008_1_ (Applied Biosystems, Foster City, CA, USA). The Applied Biosystems 7900HT Real-Time Polymerase Chain Reaction System was used for detection of SNP. The cycling program started with heating at 95°C for 10 min, followed by 40 cycles (at 95°C for 15 s and at 60°C for 1 min). Allelic discrimination was done using the software of Applied Biosystems.

### Laboratory analyses and anthropometric measurement

Lipid measurements were done for reference group subjects only. Blood samples were taken in the morning after fasting at least 12 hours. Total serum cholesterol (TChol), LDL-Chol, HDL-Chol, and triglyceride (TG) levels were determined by automatic analyzer by conventional enzymatic methods in a certified laboratory.

The height of participants (without shoes) was measured with an accuracy of one centimeter, using a stadiometer. The body weight of participants, wearing light indoor clothing and no shoes, was measured with an accuracy of 0.1 kg, using standardized medical scales. Body mass index (BMI) was calculated as weight divided by height squared (kg/m^2^).

### Statistical methods

The data were analyzed with the statistical software package SPSS version 19.0 for Windows. Analyses were performed separately for men and women. Categorical variables were expressed as proportion. Continuous variables were presented as mean values and standard error (SE).

Statistical analysis using a general linear model (GLM) was conducted to test the effects of SCARB1 genotype interaction on TChol, LDL-Chol, HDL-Chol, TG levels. This model also included age and BMI as continuous variables. The SCARB1 genotype rates in the male and female age groups were compared using Fisher’s exact test. Relationships were considered to be statistically significant when p < 0.05.

Multivariable binary logistic regression analysis was performed to identify the effect of SCARB1 genotype TT *vs.* the dual SCARB1 genotypes, CC and CT, with the risk of an MI in consideration of age, BMI, expressed as odds ratios (OR) and 95% confidence intervals (CI). Independent variables including age and BMI were entered into the models as continuous variables. P values < 0.05 were considered to be statistically significant. Multivariable logistic regression analysis was performed separately for men and women in all three age groups (25–44, 45–64, 65–74).

### Ethics statement

The study protocol was approved by the Lithuanian Bioethics Committee (Protocol No.BE-2-28; No.05/09). Written informed consent for the participation in the study was obtained from all participants.

## Abbreviations

SCARB1: Scavenger receptor Class B Type 1 gene; SNP: Single nucleotide polymorphism; CAD: Coronary artery disease; MI: Myocardial infarction; HDL-Chol: High density lipoprotein cholesterol; LDL: Low density lipoprotein; VLDL: Very low density lipoprotein; TChol: Total cholesterol; TG: Triglyceride; BMI: Body mass index; SE: Standard error; GLM: General linear model; OR: Odds ratio; CI: Confidence intervals

## Competing interests

None of the authors has any proprietary interests or conflicts of interest related to this submission. This submission has not been published anywhere previously, and it is not simultaneously being considered for any other publication.

## Authors’ contributions

DS participated in the design of the study, has made analysis and interpretation of data; drafted the manuscript. VL has made substantial contributions to conception and design, revised manuscript critically for important intellectual content, has given final approval of the version to be published. DZ has made substantial contributions to conception and design, helped to draft the manuscript. AS carried out the immunoassays, participated in the sequence alignment. OG has made acquisition of data, coordinated study, revised manuscript critically for important intellectual content. DZP has made acquisition of data, participated in the design of the study. AT has made substantial contributions to conception and design, coordinated study, participated in acquisition of data, revised manuscript critically for important intellectual content. DL has made analysis and interpretation of data; performed the statistical analysis, helped to draft the manuscript. JP has made substantial contributions to conception and design, coordinated study, revised manuscript critically for important intellectual content. RZ has made substantial contributions to conception and design, revised manuscript critically for important intellectual content. All authors read and approved the final manuscript.
